# Roadmap to personalized medicine

**DOI:** 10.3325/cmj.2012.53.294

**Published:** 2012-08

**Authors:** Malak Qattan, Constantinos Demonacos, Marija Krstic-Demonacos

**Affiliations:** 1 Faculty of Life Sciences, The University of Manchester, Manchester, UK *Marija.Krstic-Demonacos@manchester.ac.uk*; 2 School of Pharmacy, The University of Manchester, Manchester, UK

## Abstract

**Abstract:**

Standard clinical protocols and the concept “one drug fits all” that are currently used to treat illness in many cases are not effective, and strikingly so in the treatment of cancer, where 75% of therapeutic schemes are ineffective. The concept of personalized medicine is that the treatment of the disease is designed on the basis of the individual needs of each patient and the factors that influence their response to different drugs. Individualization of patient care has the potential to generate novel effective therapies, limit the adverse drug effects, create optimal treatments for individual patients, and decrease the cost associated with chronic illness and complications of drug usage. However, to achieve the goals of personalized medicine many challenges must be addressed. Here we discuss possible ways to increase the consistency of data generated by basic research and their suitability for application in medicine. New technologies employing systems biology and computer based approaches will facilitate overcoming many of the scientific challenges in the field. Changes in the education of researchers, health professionals, and the public are also required to successfully implement personalized medicine as a routine in the clinic. Finally, shift of the focus away from the development of blockbuster drugs in the biopharmaceutical industry, and modifications in the legal system to accommodate novel advancements need to be considered. The joint effort of all interested parties is needed to generate an efficient roadmap that will take us rapidly and safely to effective individual treatment, which will eliminate diseases and create better health care for all.

## Personalized medicine: background and significance

The European Cooperation in Science and Technology (COST) Conference entitled “Personalised Medicine: Better Healthcare for the Future” held in June 2012, in Larnaca, Cyprus focused on identifying ways of improving patient care and outlining the required measures to further develop medical, science, and technology fields in order to provide better medicine for all (1). The diversity of the disciplines of the delegates which included biologists, mathematicians, engineers, computer scientists, chemists, clinicians, sociologists, philosophers as well as politicians, and representatives from funding agencies and pharmaceutical companies reflected the different angles from which the subject of personalized medicine was viewed.

Personalized medicine is defined as the individualized treatment tailored to the needs of a particular patient and not based only on the type of their illness. The aim of personalized medicine is to design appropriate treatment for each person's unique needs, taking into account clinical, biological, genetic, environmental, and socioeconomic factors and life styles. This ideally should allow accurate predictions to be reached about a person's susceptibility to develop disease, response to treatment, and elimination of therapeutic failure and toxicity. Currently, many diseases are not treated successfully, the therapeutic strategies are often symptomatic, and numerous drugs are effective only for certain groups of patients (2). The effective dose of the appropriate medicine prescribed for a particular disease might vary among different individuals depending on the patient genetic constitution. Furthermore, the choice of the medicine and the effective dose might be different for the same individual at different stages of their life. Although selective toxicity has been known for several years it has recently become evident that this is mostly attributable to the action of metabolizing enzymes such as members of the cytochrome P450 family, which alter drug metabolism and can affect pharmacodynamic parameters depending on the genetic background of the patient (3).

A few examples of stratified therapies used in the clinical practice include the case of non*-*small cell lung cancer (NSCLC), in which either the epidermal growth factor receptor (EGFR) (4) or the anaplastic lymphoma kinase (ALK) pathways are deregulated (5). Cancer patients carrying mutations in the EGFR pathway respond better to Tarceva (erlotinib), whereas the recently approved chemotherapeutic drug Xalkori (crizotinib) is more efficient in those patients bearing (ALK) gene rearrangement (6). Breast cancer patients overexpressing human epidermal growth factor receptor 2 (HER2) are treated with the antibody Herceptin (trastuzumab), which inhibits this pathway providing another promising paradigm of personalized treatment (7). Patient stratification is also employed for treatment of coagulation disorders with warfarin based on CYP2C9, vitamin K epoxide reductase complex, subunit 1 (VKORC1) and Protein C status, and international normalized ratio values determined throughout the period of the treatment (8). The treatment of metastatic melanoma with the BRAF inhibitor is dependent on the status of this kinase in the tumor (9). Although a few prominent cases of personalized medicine have recently emerged, we are still far from efficiently treating many diseases as complications due to the side effects of drugs, and the increasing costs of the therapeutic failure remain considerable.

## Challenges encountered in the field of personalized medicine

Analysis of the efficacy of numerous drugs currently used to treat major diseases shows that many patients do not respond to therapy. This could vary from 20% treated with analgesics up to 75% of cancer patients treated with chemotherapeutics (10), indicating that we are still far from knowing which genetic markers have the most clinical significance in various diseases and different individuals (11). One potential reason for the inefficiency of several therapeutic schemes is that the experimental settings currently used for biotechnology-oriented research and drug development processes may not be translated efficiently into the clinical practice. Possible ways to address this problem is to increase investment in bridging basic and translational research and foster collaborative multidisciplinary studies (12). Furthermore, information about both basic and translational research should become readily available. For example, to improve the consistency of published data, negative results that do not confirm hypothesis and data sets without the completed molecular mechanisms involved, should become acceptable for publication in high impact and open access journals linked to as many databases as possible. The increase of data availability will provide wider base for data mining, improve the modeling of diseases and therapeutic schemes, and shorten the time and cost of the drug development and clinical trials processes (13).

In addition to individual variability, many diseases are heterogeneous in terms of the underlying cause. For example, one or more of the hallmarks of cancer (14) can vary between individual tumors and arise due to oncogene activation or mutations of tumor suppressor genes, and their combined effects on each pathway might be selectively changed in a particular cell type within the tumor and hence require different pharmacological intervention. Treatment of heterogeneous populations of tumor cells with the same drug, in most cases leads to resistance, therefore continuous assessment of the evolution of the disease at the molecular level is necessary to address these issues. Generation of large logical models that incorporate many interactions facilitates the identification of potential shifts in the dependency of cancer cells from one particular signaling pathway to another during a course of treatment, thereby allowing design of alternative therapeutic protocols to avoid resistance. Finally, the stage of disease may determine the appropriate treatment as for example the levels of the CYP2E1 biomarker are high in early stages of breast cancer and gradually decline in later stages of tumor development (15,16), therefore stratifying patients according to the CYP2E1 cellular levels.

The vast amount of information that has accumulated throughout recent years of basic and clinical research has made it difficult to analyze the data collected so far using current methodologies. Novel systems biology and text mining approaches coupled with bioinformatics and innovative modeling techniques are of crucial importance for progress in the field. The assembly of a multidisciplinary team including biologists, physicists, and clinicians was the only way to successfully address complicated issues we encountered in some of our studies (17,18).

It has become clear that successful implementation of personalized treatment requires changes in the education of future clinicians, health professionals, and basic scientists to include fundamental elements of pharmacogenetics and pharmacogenomics. In addition, confidence in the efficiency of individualized medicines will increase if information about advancements in the field is conveyed to the patients and the public. Novel pilot schemes and training programs for health professionals are in development in France (19). It will be necessary to expand these types of educational programs that include systems and synthetic biology and predictive modeling approaches in order to prepare health professionals and other members of future interdisciplinary teams for participation in collaborative research projects and management of personalized therapies. More has to be done toward refining the legal frame regarding privacy issues that individual treatment may bring. Who will be allowed to have access to sensitive patients’ data? How access to these data could be restricted to those professionals whose primary interest is the benefit to the patients’ health? It is essential that potential adverse influence of this information on employability, life and health insurance policies, and other areas of everyday life is prevented. Opportunities must be explored for more extensive public input and confidence in the notion that the routine use of personalized medicine will lead to real health improvements.

Multidisciplinary and collaborative research is the way forward to overcome barriers we all encounter in our research efforts due to lack of communication or understanding that is required to cross the boundaries into different disciplines. Successful collaborations usually emerge as a result of personality compatibilities between collaborators and not overlap in their scientific expertise, thus they are often left to chance. This is not sufficient to fulfill the expectations of innovative research in the future as multidisciplinary teams and collaborative efforts are essential prerequisites for success toward realizing the goals of personalized medicine. Therefore, we propose that we place in action mechanisms to facilitate collaboration and multidisciplinary approaches such as awarding researchers and clinicians with promotions and further research funds to encourage these efforts. COST has already fostered many of these successful events that facilitated collaborative efforts and multidisciplinary approaches but more needs to be done toward these goals to involve other funding bodies and institutions (20). For example, majority of cases for promotions at Universities, research institutions, and hospitals are judged on individual’s own contribution to scientific progress as an independent researcher, whereas collaborative efforts are mostly viewed as lower impact contribution.

Finally, the question whether personalized medicine will be cost-effective and how it will be funded has not yet been clearly answered. Why do we need basic, curiosity driven, “blue sky” research and especially now in a period of financial crisis? How can funding of something the public does not understand be of benefit? Should we fund only applied research?? The wider population should become aware of the benefits of funding basic research and multidisciplinary approaches as progress in science is the only way forward.

## Conclusions and perspectives

Diagnosis and treatment of illness are currently performed on the basis of clinical protocols designed for a particular disease and not a particular patient. However, given the low efficacy of numerous drugs, the high cost of their development, and the price national health systems pay for the management of chronic patients and complications of drug treatments, the urgent need to shift the focus from designing one drug that fits all toward the research that actively pursues the goals of personalized medicine is becoming apparent.

Taking this path has many challenges and complexities. The way that knowledge accumulated through basic science research is transferred to drug development companies and general public must change to involve more transparent publication processes and include reporting negative data. Changes in researchers’ career progression and the way their work is judged need to be considered. Novel technologies addressing the heterogeneity of responses to drugs not only among different individual patients but in one patient affecting the response to treatment should be embraced. Technological advances in sequencing resulted in accumulation of vast data sets that need to be analyzed by novel mining tools and require changes in education of researchers and health professionals, as well as changes in guidelines and legislation provided by the regulatory authorities. Finally, funding strategies seem to shift toward interdisciplinary teams but we must keep basic research funding strategies as well as providing ways of successfully fostering collaboration.

## References

[1] Louca S, Pochet R, Schinzer D (2012). Why another conference on personalized medicine?. Croat Med J.

[2] Ginsburg GS, McCarthy JJ (2001). Personalized medicine: revolutionizing drug discovery and patient care.. Trends Biotechnol.

[3] Pinto N, Dolan ME (2011). Clinically relevant genetic variations in drug metabolizing enzymes.. Curr Drug Metab.

[4] Chen G, Kronenberger P, Teugels E, Umelo IA, De Greve J (2012). Targeting the epidermal growth factor receptor in non-small cell lung cancer cells: the effect of combining RNA interference with tyrosine kinase inhibitors or cetuximab.. BMC Med.

[5] Chiarle R, Voena C, Ambrogio C, Piva R, Inghirami G (2008). The anaplastic lymphoma kinase in the pathogenesis of cancer.. Nat Rev Cancer.

[6] Shaw AT, Solomon B (2011). Targeting anaplastic lymphoma kinase in lung cancer.. Clin Cancer Res.

[7] Spector NL, Blackwell KL (2009). Understanding the mechanisms behind trastuzumab therapy for human epidermal growth factor receptor 2-positive breast cancer.. J Clin Oncol.

[8] Sconce EA, Khan TI, Wynne HA, Avery P, Monkhouse L, King BP (2005). The impact of CYP2C9 and VKORC1 genetic polymorphism and patient characteristics upon warfarin dose requirements: proposal for a new dosing regimen.. Blood.

[9] Ades F, Metzger-Filho O. (2012). Targeting the cellular signaling: BRAF inhibition and beyond for the treatment of metastatic malignant melanoma. Dermatol Res Pract.

[10] Spear BB, Heath-Chiozzi M, Huff J (2001). Clinical application of pharmacogenetics.. Trends Mol Med.

[11] Karas-Kuzelicki N, Mlinaric-Rascan I (2009). Individualization of thiopurine therapy: thiopurine S-methyltransferase and beyond.. Pharmacogenomics.

[12] Louca S (2012). Personalized medicine – a tailored health care system: challenges and opportunities.. Croat Med J.

[13] Henney AM (2012). The promise and challenge of personalized medicine: aging populations, complex diseases, and unmet medical need.. Croat Med J.

[14] Hanahan D, Weinberg RA (2011). Hallmarks of cancer: the next generation.. Cell.

[15] Leung T, Alavi H, Andreou K, Krstic-Demonacos M, Demonacos C (2011). The role of cytochromes P450 in cancer and their potential as targets for anti-cancer drug development.. Int J Mol Med.

[16] Vaclavikova R, Hubackova M, Stribrna-Sarmanova J, Kodet R, Mrhalova M, Novotny J (2007). RNA expression of cytochrome P450 in breast cancer patients.. Anticancer Res.

[17] Chen DW, Lynch JT, Demonacos C, Krstic-Demonacos M, Schwartz JM (2010). Quantitative analysis and modeling of glucocorticoid-controlled gene expression.. Pharmacogenomics.

[18] Chen DW, Liu JZ, Saha V, Schwartz JM, Krstic-Demonacos M (2012). Erg and AP-1 as determinants of glucocorticoid response in acute lymphoblastic leukemia.. Oncogene.

[19] Haiech J, Kilhoffer MC (2012). Personalized medicine and education: the challenge.. Croat Med J.

[20] Duburs G, Neibecker D, Zarkovic N (2012). Chemistry and personalised medicine – the research and development future of Europe.. Croat Med J.

